# Association Between Cardiovascular Risk Factors and the Progression of Motor and Non-Motor Symptoms in Parkinson’s Disease: A Five-Year Cohort Study

**DOI:** 10.3390/jcm15093217

**Published:** 2026-04-23

**Authors:** Junyi Chen, Jing Chen, Zhe Zhao, Danhua Zhao, Baoyu Chen, Qi Wang, Yuan Li, Chaobo Bai, Xintong Guo, Jinjin Wang, Junliang Yuan

**Affiliations:** 1 Department of Neurology, Peking University Sixth Hospital, Peking University Institute of Mental Health, NHC Key Laboratory of Mental Health (Peking University), National Clinical Research Center for Mental Disorders (Peking University Sixth Hospital), Beijing 100191, China; 2 Department of Pharmacy, Peking University Third Hospital, Beijing 100191, China

**Keywords:** cardiovascular risk factors, Parkinson’s disease, motor symptoms, non-motor symptoms

## Abstract

**Objective**: To investigate the relationship between cardiovascular risk factors and the progression of motor and non-motor symptoms in Parkinson’s disease (PD). **Methods**: We used data from the Parkinson’s Progression Markers Initiative (PPMI) cohort with a follow-up duration of >5 years. Baseline assessments included genetic analysis, brain MRI, cardiovascular risk factors, and overall cardiovascular disease (CVD) risk. Motor symptoms and non-motor symptoms of PD were evaluated using the Movement Disorders Society revised Unified Parkinson’s Disease Rating Scale (MDS-UPDRS) and sub-scores, Hoehn–Yahr stage, and Montreal Cognitive Assessment (MoCA). Statistical analyses comprised univariate and multivariate linear regression and stratified analysis. **Results**: A total of 169 newly diagnosed PD patients and 78 healthy controls (HCs) were included. At baseline, no significant differences in cardiovascular risk factors or overall CVD risk were observed between PD patients and HCs. Hypertension (*β* = 6.748, *p* = 0.040) and hyperlipidemia (*β* = 8.316, *p* = 0.005) were associated with faster motor progression. *ApoE* genotype was correlated with motor progression (*β* = 7.593, *p* = 0.007). PD patients with a moderate-to-low CVD risk (<20%) had milder axial motor symptoms (3.0 [IQR, 4.0] vs. 4.0 [IQR, 5.0], *p* = 0.048) and lower MDS-UPDRS Part I total scores (7.0 [IQR, 6.25] vs. 9.0 [IQR, 7.0], *p* = 0.039) at last follow-up compared to high-CVD-risk (≥20%) patients. Overall CVD risk was negatively correlated with total MoCA score at last follow-up (*β* = −0.208, *p*< 0.001). **Conclusions**: Cardiovascular risk factors accelerate the progression of motor and non-motor symptoms in PD, suggesting that management of modifiable CVD risk factors may represent a promising target to delay the progression of PD.

## 1. Introduction

Parkinson’s Disease (PD) is the second most prevalent neurodegenerative disorder in the world, following Alzheimer’s disease, and has emerged as the fastest-growing neurological condition worldwide. Projections indicate that the global prevalence of PD will exceed 25 million individuals by 2050 [1,2], imposing a substantial socioeconomic and healthcare burden. Clinically, PD is characterized by a combination of motor and non-motor symptoms. Cardinal motor manifestations include resting tremor, bradykinesia, rigidity, and postural instability with gait disturbances (PIGDs). Non-motor symptoms, such as constipation, olfactory dysfunction, sleep disorders, autonomic dysfunction, cognitive impairment, and psychiatric symptoms [1,3,4,5], often precede motor onset or worsen with disease progression and are associated with poor prognosis. These non-motor features are strongly associated with reduced health-related quality of life and increased caregiver burden. To date, no definitively effective disease-modifying therapies have yet been approved for PD; current interventions focus solely on symptomatic management without evidence of slowing the progression of PD. Neurodegeneration in PD is driven by a complex interplay of multiple pathogenic mechanisms, including α-synuclein aggregation, neuroinflammation, mitochondrial dysfunction, and metabolic dysregulation [3,5]. Recent evidence has highlighted glucose metabolism abnormalities as a key contributor to PD pathogenesis: recurrent hypoglycemic episodes and glycemic variability have been associated with accelerated dopaminergic neuronal loss and worse clinical outcomes in PD patients, even in the absence of pre-existing diabetes [6]. These metabolic disturbances may contribute to both the initiation of neurodegenerative processes and exacerbate existing pathological changes, highlighting the bidirectional relationship between systemic metabolic factors and PD progression.

Cardiovascular disease (CVD) is the leading cause of global mortality [7]. Accumulating evidence has identified shared risk factors between PD and CVD, including advanced age, male sex, and type 2 diabetes mellitus. Conversely, caffeine consumption and regular physical activity are recognized as common protective factors for both conditions [8], implying potential overlap in their pathogenic mechanisms. Genome-wide association studies (GWASs) have revealed shared genetic risk loci and regulatory pathways between PD and CVD, particularly those involved in inflammation, mitochondrial dysfunction, and cellular metabolism [9,10]. Clinically, the two diseases also frequently co-occur; however, their bidirectional relationship remains poorly understood. While some studies suggest that CVD risk factors elevate PD risk and that PD patients have increased CVD risk [11,12], others report no significant difference or even a reduced risk relative to controls [13]. Similarly, investigations into the association between CVD risk factors and PD motor symptom progression have yielded conflicting results. A cross-sectional study from the USA found a correlation between CVD risk factors and axial motor symptoms in PD [14], whereas a cohort study conducted in the USA and UK observed no link between CVD risk factors and motor symptom progression [15]. Basic preclinical research has suggested that statins may exert disease-modifying effects on PD [16]; yet, a recent randomized controlled trial (RCT) demonstrated that simvastatin was ineffective as a disease-modifying therapy for patients with moderate-to-severe PD [17]. Thus, the potential impact of interventions targeting CVD risk factors or CVD treatment on PD onset or progression remains uncertain.

This study aimed to investigate the associations between individual cardiovascular risk factors, overall CVD risk, and the progression of motor and non-motor symptoms in newly diagnosed PD patients from the Parkinson’s Progression Markers Initiative (PPMI) cohort.

## 2. Methods

### 2.1. Study Design and Participants

Data for this study were downloaded from the PPMI database [18] on 1 October 2024. PPMI is an ongoing comprehensive observational, longitudinal, international, multicenter study designed to identify PD progression biomarkers. Details including eligibility criteria and methods of PPMI and all study data are available on the PPMI website (www.ppmi-info.org) (accessed on 1 October 2024). All participants in the PPMI provided written informed consent, and the PPMI study was approved by the institutional review board at each study site. In brief, participants include patients with newly diagnosed PD (diagnosed within 2 years at screening visit) and healthy controls (HCs). At baseline, PD patients were required to (1) have an asymmetric resting tremor or asymmetric bradykinesia or two of bradykinesia, resting tremor, and rigidity, (2) be diagnosed within 2 years and untreated for PD, (3) have a dopamine transporter (DAT) deficit on DAT imaging, (4) be over 30 years of age. HCs must have no current or active clinically significant neurological disorder, no first-degree relative with PD, a Montreal Cognitive Assessment (MoCA) total score > 26, and normal DAT imaging results as confirmed by visual inspection. After enrollment, participants were assessed every 3 months during the first year and every 6 months thereafter. We collected data on motor and non-motor symptoms at both baseline and the last follow-up visit. Notably, all participants were followed up for more than 5 years.

### 2.2. Assessment of Demographic and Clinical Characteristics

We assessed various demographic and clinical characteristics, including age at baseline, sex, years of education, family history of PD, disease duration at baseline (time from symptom onset to baseline), time from PD diagnosis to baseline, and levodopa equivalent daily dose (LEDD) at baseline and the last follow-up visit. LEDD was calculated based on the records of dopaminergic treatment and previous guidelines [19]. Genetic analysis was performed on each participant. *ApoE* genotype and coding variants for PD pathogenic genes (*LRRK2*, *GBA*, *VPS35*, *SNCA*, *PRKN*, *PARK7*, and *PINK1*) were included in our study. *ApoE* genotype was categorized as *ApoE* ε4 status (ε4 homozygous, heterozygous, or negative) and the number of pathogenic variants of the above PD-related genes was counted. At baseline, each participant underwent a brain Magnetic Resonance Imaging (MRI) scan. The MRI results were categorized into three different groups: normal findings; abnormal findings without clinical significance; and abnormal findings with clinical significance.

### 2.3. Assessment of Cardiovascular Risk Factors

Medical history, smoking status, height, weight, and blood pressure of all participants at baseline were collected from the PPMI database. Diagnoses of type 2 diabetes mellitus, hypertension, hyperlipidemia (elevated serum total cholesterol, low-density lipoprotein cholesterol [LDL-C], or triglycerides), gout, CVD, cerebral vascular disease, and peripheral vascular disease were extracted from the participants’ medical history. CVD was defined as a documented history of coronary artery disease, myocardial infarction, angina pectoris, coronary angioplasty and stents, or coronary artery bypass grafting. Cerebral vascular disease was defined as a documented history of stroke, transient ischemic attack, or transient global amnesia. Body mass index (BMI) was calculated using height and weight. Obesity was defined as a BMI ≥ 30 kg/m^2^ [20]. Participants with a smoking history were defined as having smoked 100 or more cigarettes during their lifetime. Furthermore, overall cardiovascular risk was assessed using three indicators: CVD risk points, CVD risk, and CVD risk ratio. Since serum LDL-C levels were unavailable in the PPMI dataset, CVD risk points were calculated using a simplified model of the Framingham risk score (FRS), incorporating non-laboratory predictors including age, sex, BMI, diabetes diagnosis, systolic blood pressure, and antihypertensive medication [21]. This sex-stratified simplified model adopts BMI as a substitute for cholesterol in the predictor set, and follows exactly the same modeling protocols and performance evaluation frameworks as those used in the development of the FRS, and performs reasonably well in predicting 10-year CVD risk [21]. CVD risk was determined based on the CVD risk points to estimate an individual’s 10-year risk of CVD. Participants were stratified into three groups according to their CVD risk: low-risk group (<10%), moderate-risk group (10–20%), and high-risk group (>20%) [22,23]. The CVD risk ratio was defined as the ratio of an individual’s CVD risk points to the normative CVD risk points (calculated by setting all CVD risk factor parameters within the normal range for a given individual’s age and sex). A CVD risk ratio > 1.0 indicated that the participants had a higher CVD risk than individuals of the same age and sex.

### 2.4. Assessment of Motor and Non-Motor Symptoms

Motor symptoms of participants were assessed using the Movement Disorders Society revised Unified Parkinson’s Disease Rating Scale (MDS-UPDRS). Motor experiences of daily living were evaluated using the total score of MDS-UPDRS Part II (UPDRS II). Motor progression was evaluated by analyzing changes in the MDS-UPDRS Part III (UPDRS III) scores from baseline to the last follow-up visit. The severity of motor symptoms was gauged according to the Hoehn and Yahr stages. Motor subtypes, namely tremor-predominant (TD), PIGD-predominant, and indeterminate, were classified based on baseline UPDRS II and UPDRS III scores with the use of published methods [24]. The total score and sub-scores of UPDRS III were analyzed independently. Individual motor symptoms were assessed using specific items from UPDRS III. For tremor, items included 3.15–3.18; for rigidity, it was item 3.3; for bradykinesia, the items were 3.4–3.8 and 3.14; for PIGD, the items were 3.10–3.12. A 5-item axial motor sub-score was computed by summing items 3.1, 3.9, 3.10, 3.12, and 3.13 from the UPDRS III examination [14]. Motor complications were identified using MDS-UPDRS Part IV (UPDRS IV). All patients were evaluated separately in “on” and “off” states at the last follow-up visit.

Non-motor experiences of daily living were evaluated using the total score of MDS-UPDRS Part I (UPDRS I). Individual non-motor symptoms were assessed using specific items from MDS-UPDRS Part I. For psychotic symptoms, it was item 1.2; for mood complaints, the items included 1.3–1.5; for sleep disorders, the items were 1.7 and 1.8; for autonomic symptoms, the items were 1.10 and 1.11. Global cognition was assessed with MoCA.

### 2.5. Statistical Analysis

We analyzed the data collected at baseline and the last follow-up visit. The Shapiro–Wilk test was used to assess the normality of continuous variables. Normally distributed continuous variables were presented as mean ± standard deviation (SD), and non-normally distributed variables as median (interquartile range, IQR). Categorical variables were shown as frequencies and percentages. For group comparisons of continuous variables, Student’s *t*-test was used for normally distributed data and the Mann–Whitney *U* test was used for non-normally distributed data. For categorical variables, the chi-square test or Fisher’s exact test was applied.

Independent variables included: age, sex, PD family history, years of education, smoking status, disease duration, *ApoE* genotype, count of pathogenic variants, brain MRI result, BMI, systolic blood pressure, diastolic blood pressure, type 2 diabetes mellitus, hyperlipidemia, hypertension, history of cardiovascular and cerebrovascular diseases, gout, obesity, CVD points, CVD risk, and CVD risk ratio. Dependent variables, including the total score of UPDRS II, Hoehn–Yahr stage, the total scores of UPDRS III, motor subtypes, tremor sub-score, rigidity sub-score, bradykinesia sub-score, PIGD sub-score, axial motor sub-score, the total score of UPDRS IV, the total score of UPDRS I, psychotic symptom sub-score, mood complaint sub-score, sleep disorder sub-score, autonomic symptom sub-score, the total scores of MoCA, and LEDD, were related to the motor and non-motor symptoms of PD. Baseline and last follow-up data of the dependent variables were analyzed separately. Since this study enrolled a cohort of newly diagnosed PD patients, all participants had a LEDD of 0 at baseline. Therefore, only LEDD at last follow-up was included. Motor-related scores were evaluated in both “on” and “off” states at last follow-up visit. The differences between the total UPDRS III scores and axial motor sub-scores at last follow-up (both “on” and “off” states) and their corresponding baseline values were calculated. These differences, which are defined as the change in total UPDRS III score and change in axial motor sub-score (for both “on” and “off” states), were used as dependent variables. Univariate linear regression analysis was performed to examine the relationships between each independent variable and the dependent variables at both baseline and last follow-up, with Bonferroni correction for multiple comparisons. Subsequently, multivariate linear regression analysis was conducted to further validate the associations between cardiovascular risk factor-related independent variables and each dependent variable. Standardized effect sizes equivalent to Cohen’s d were calculated for all predictors in the multivariable linear regression models. All effect sizes were interpreted using standard conventions: small (d ≥ 0.2), moderate (d ≥ 0.5), and large (d ≥ 0.8).

We stratified patients with PD into two groups based on baseline CVD risk: high CVD risk (≥20%) and moderate-to-low CVD risk (<20%). Then we compared differences in motor and non-motor symptoms between the two groups at baseline and last follow-up visit. A *p* value < 0.05 was considered statistically significant. All analyses were performed using R (version 4.4.3).

## 3. Results

### 3.1. The Clinical Characteristics of the Participants at Baseline

A total of 169 newly diagnosed PD patients and 78 HCs were enrolled in this study. All participants completed a follow-up period of >5 years, with a median follow-up duration of 9 years. The baseline demographic and clinical characteristics of the study cohort are described in Table 1. The mean age of PD patients was 61.7 ± 8.1 years, and that of HCs was 60.0 ± 10.2 years. There were no significant differences between the two groups in terms of age, sex, years of education, smoking status, diabetes mellitus, hyperlipidemia, hypertension, history of cardiovascular and cerebrovascular diseases, obesity, gout, brain MRI, *ApoE* genotype, MoCA scores, CVD risk points, or CVD risk ratios (*p* > 0.05). In contrast, significant differences were found between PD patients and HCs for PD family history, PD pathogenic gene variant carrier status, CVD risk, and UPDRS III scores (*p* < 0.05).

### 3.2. Association Between CVD Risk Factors and Motor and Non-Motor Symptoms in PD Patients

Firstly, univariate linear regression analysis was performed, and the relationships of CVD risk points, CVD risk, and CVD risk ratio with motor and non-motor symptoms in PD patients are presented in Table 2 and Table 3. For motor symptoms, the CVD risk points were correlated with the baseline total UPDRS III score (*β* = 0.305, *p* = 0.049), baseline tremor sub-score (*β* = 0.142, *p* = 0.014), total UPDRS III score in “on” state at last follow-up (*β* = 0.596, *p* = 0.011), tremor sub-score in “on” state at last follow-up (*β* = 0.116, *p* = 0.047), and bradykinesia sub-score in “on” state at last follow-up (*β* = 0.278, *p* = 0.020). They were also correlated with the axial motor sub-score at baseline (*β* = 0.082, *p* = 0.002), in “on” state at last follow-up (*β* = 0.143, *p* = 0.009), and in “off” state at last follow-up (*β* = 0.136, *p* = 0.024). The CVD risk ratio was correlated with the baseline tremor sub-score (*β* = 1.736, *p* = 0.017), total UPDRS II score at last follow-up (*β* = 3.529, *p* = 0.026), and LEDD at last follow-up (*β* = 243.356, *p* = 0.024). However, no statistically significant associations were observed between CVD risk points, CVD risk, or CVD risk ratio and PD motor symptoms after Bonferroni correction.

For non-motor symptoms, the CVD risk points were correlated with the baseline sleep disorder sub-score (*β* = 0.061, *p* = 0.016), baseline autonomic dysfunction sub-score (*β* = 0.048, *p* = 0.007), total UPDRS I score at last follow-up (*β* = 0.197, *p* = 0.038), and total MoCA score at baseline (*β* = −0.134, *p* < 0.001) and at last follow-up (*β* = −0.208, *p* < 0.001). CVD risk was correlated with the baseline total UPDRS I score (*β* = −1.472, *p* = 0.027) and baseline autonomic dysfunction sub-score (*β* = −0.380, *p* = 0.036). However, only the association between CVD risk points and the total MoCA score at last follow-up remained significant after Bonferroni correction (adjusted *p* = 0.048). No significant associations were observed between CVD risk points, CVD risk, or CVD risk ratio and other non-motor symptoms of PD.

Additionally, univariate linear regression revealed that age correlated with total MoCA score at last follow-up after Bonferroni correction (*β* = −0.136, *p* < 0.001). PD duration correlated with baseline sleep disorder sub-score (*β* = 0.382, *p* < 0.001), baseline psychotic symptom sub-score (*β* = 0.046, *p* < 0.001), and baseline total UPDRS IV (*β* = 0.566, *p* < 0.001); and the number of pathogenic variants in PD-related genes correlated with baseline sleep disorder sub-score (*β* = 1.002, *p* < 0.001).

**Figure 1:**
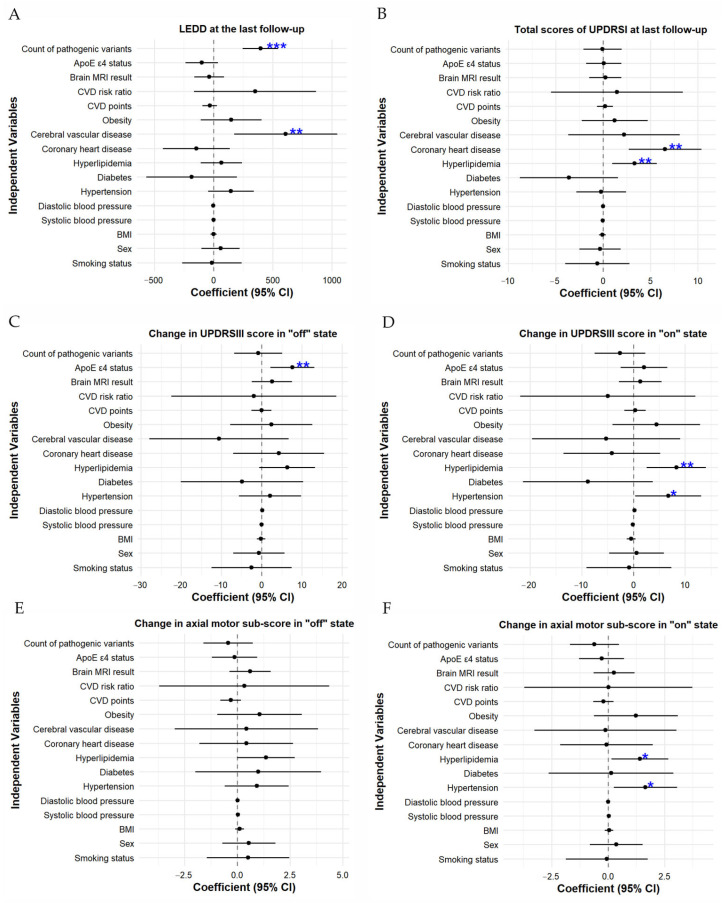
Forest plot of the associations between independent variables and dependent variables: (**A**). LEDD at last follow-up, (**B**). Total UPDRS I score at last follow-up, (**C**). Change in UPDRS III score in “off” state, (**D**). Change in UPDRS III score in “on” state, (**E**). Change in axial motor sub-scores in “off” state, (**F**). Change in axial motor sub-scores in “on” state. * *p* < 0.05, ** *p* < 0.01 and *** *p* < 0.001.

In our study, multivariate linear regression was also performed with cardiovascular risk factors and follow-up duration as independent variables and PD symptoms as dependent variables. This analysis showed good model fit (*R*^2^ > 0.2, *p* < 0.05) for the following indices: last follow-up LEDD, last follow-up total UPDRS I, change in UPDRS III scores in “on” and “off” state, and change in axial motor scores in “on” and “off” state. Specifically, the number of pathogenic variants in PD-related genes (*β* = 397.071, *p* < 0.001, Cohen’s d = 0.76, moderate effect) and history of cerebrovascular disease (*β* = 609.877, *p* = 0.007, Cohen’s d = 1.17, large effect) correlated with last follow-up LEDD; hyperlipidemia correlated with changes in UPDRS III score in “on” state (*β* = 8.316, *p* = 0.005, Cohen’s d = 0.55, moderate effect), changes in axial motor sub-score in “on” state (*β* = 1.413, *p* = 0.030, Cohen’s d = 0.44, moderate effect), and last follow-up total UPDRS I (*β* = 3.304, *p* = 0.007, Cohen’s d = 0.57, moderate effect); hypertension correlated with changes in UPDRS III score in “on” state (*β* = 6.748, *p* = 0.040, Cohen’s d = 0.45, moderate effect) and changes in axial motor sub-score in “on” state (*β* = 1.655, *p* = 0.022, Cohen’s d = 0.51, moderate effect); *ApoE* genotype correlated with change in UPDRS III score in “off” state (*β* = 7.593, *p* = 0.007, Cohen’s d = 0.44, moderate effect); and history of cardiovascular disease correlated with last follow-up total UPDRS I score (*β* = 6.532, *p* = 0.001, Cohen’s d = 1.12, large effect). No significant associations were observed between sex, smoking status, diabetes, CVD risk points, CVD risk, CVD risk ratio, or other cardiovascular risk factors and the aforementioned PD symptom indices; details are available in Figure 1.

### 3.3. Comparison of Clinical Characteristics Between High and Moderate-to-Low-CVD Risk PD Patients

Based on baseline CVD risk, PD patients were stratified into a moderate-to-low-CVD-risk subgroup (CVD risk < 20%) and a high-CVD-risk subgroup (CVD risk ≥ 20%), with their baseline demographic and clinical characteristics detailed in Table 4. Statistically significant differences in sex (male: 42.3% vs. 70.1%, *p* = 0.001), age (57.9 ± 6.8 vs. 63.3 ± 8.1 years, *p* < 0.001), smoking status (smokers: 19.2% vs. 39.3%, *p* = 0.017), CVD risk points (12 ± 4.3 vs. 16 ± 6.0, *p* < 0.001), and CVD risk ratio (1.21 ± 0.4 vs. 1.33 ± 0.4, *p* = 0.035) were observed between the moderate-to-low- and high-CVD-risk subgroups. No significant differences were found in education, blood pressure, BMI, hyperlipidemia, diabetes mellitus, hypertension, history of cardiovascular/cerebrovascular disease, obesity, brain MRI, or *ApoE* genotype (*p* > 0.05).

**Figure 2:**
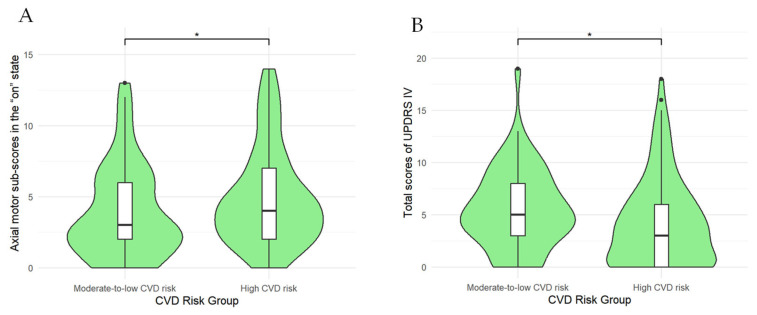
Comparisons of axial motor sub-scores in the “on” state (**A**) and total scores of UPDRS IV (**B**) at last follow-up between moderate-to-low- and high-CVD-risk PD patient subgroups. * *p* < 0.05.

**Figure 3:**
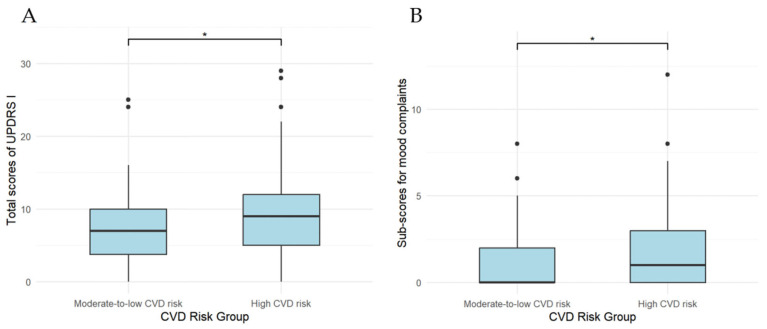
Comparisons of total scores of UPDRS I (**A**) and sub-scores for mood complaints (**B**) at last follow-up between moderate-to-low- and high-CVD-risk PD patient subgroups. * *p* < 0.05.

Indices related to motor and non-motor symptoms of PD at baseline and last follow-up in the two subgroups are detailed in Table 5 and Table 6. For motor symptoms, at last follow-up, the moderate-to-low-CVD-risk subgroup had a significantly lower axial motor sub-score in “on” state (3.0 [IQR, 4.0] vs. 4.0 [IQR, 5.0], *p* = 0.048) and a significantly higher total UPDRS IV score (5.0 [IQR, 5.0] vs. 3.0 [IQR, 6.0], *p* = 0.018) than the high-CVD-risk subgroup, as shown in Figure 2. No significant differences were observed in other motor symptoms between the two subgroups (*p* > 0.05). For non-motor symptoms, at last follow-up, the moderate-to-low-CVD-risk subgroup had significantly lower total UPDRS I scores (7.0 [IQR, 6.25] vs. 9.0 [IQR, 7.0], *p* = 0.039) and mood complaint sub-scores (0 [IQR, 2.0] vs. 1.0 [IQR, 3.0], *p* = 0.021) than the high-CVD-risk subgroup, as shown in Figure 3.

## 4. Discussion

Our study revealed no significant differences in CVD risk factors or overall CVD risk between newly diagnosed PD patients and HCs at baseline. Hypertension and hyperlipidemia were associated with faster motor progression in PD patients, especially axial motor symptoms, over a median follow-up of 8.2 years. A history of cerebrovascular disease was positively correlated with LEDD. Higher CVD risk was correlated with more severe motor and non-motor symptoms in PD, including axial motor symptoms, cognitive impairment, and affective disorders. In addition, *ApoE* genotype was associated with motor progression in PD.

The association between PD and CVD risk factors has long been controversial. The Luxembourg Parkinson’s Disease Cohort study, which enrolled 676 PD patients and 874 non-PD controls, demonstrated that individuals with a history of CVD had a significantly higher risk of PD onset [12]. A study based on the U.S. National Health and Nutrition Examination Survey (NHANES) database showed that PD was significantly associated with increased CVD mortality in U.S. adults [11]. However, an Israeli PD cohort study, which included 10,840 newly diagnosed PD patients and had a mean follow-up duration of 16.3 years, found that PD patients had a 70–80% lower CVD risk compared with the general population [13]. In the present study, newly diagnosed PD patients showed no significant differences in CVD risk factors or overall CVD risk at baseline compared with HCs. This finding may be attributed to two factors. Firstly, there were no statistically significant differences in age and sex between the HCs and PD patients. Secondly, the newly diagnosed PD patients had a short disease duration at baseline and had not yet initiated anti-PD treatment.

The association between cardiovascular risk factors and symptom progression in PD also remains controversial. An American cross-sectional study showed that overall CVD risk is associated with the progression of axial motor symptoms in PD, independent of striatal dopaminergic degeneration [14]. However, this study had a small sample size, including only 85 PD patients. Another study based on the PPMI cohort and the UK Tracking Parkinson’s cohort found no clinically significant effects of CVD risk factors and overall CVD risk on motor or cognitive progression in PD [15]. Nonetheless, this study excluded smoking status from its CVD risk analysis and had a relatively short follow-up duration. Our results suggested that hypertension and hyperlipidemia were associated with faster motor progression in PD patients, particularly axial motor symptoms. Additionally, higher overall CVD risk correlated with more severe motor and non-motor symptoms in PD, including axial motor symptoms, cognitive impairment, and affective disorders. These results were consistent with previous findings [12,14]. CVD risk factors may influence PD progression through multiple mechanisms, including cerebrovascular disease, inflammatory responses, oxidative stress, lipid metabolism disorders, and mitochondrial dysfunction.

We found that hyperlipidemia may be associated with faster axial motor symptom progression in PD. However, due to limitations of the PPMI database, LDL-C was not included in the analysis. Little is known about the association between serum total cholesterol or a history of hypercholesterolemia and PD risk. Only a few studies have examined this association, and their results are inconsistent: some epidemiological studies indicate that higher serum cholesterol levels are associated with an increased incidence of PD [25], while others report a decreased risk or no association [26,27]. Notably, cholesterol itself or cholesterol-carrying lipoproteins cannot cross the blood–brain barrier (BBB). In recent years, studies have suggested that cholesterol oxidation product 27-hydroxycholesterol may cross the BBB and regulate PD onset and progression [28]. Interestingly, another finding of our study is that the *ApoE* genotype was significantly associated with motor progression in PD patients: those carrying the ε4 allele exhibited faster motor symptom progression. The *ApoE* gene encodes apolipoprotein E, a multifunctional protein primarily involved in lipid transport and metabolism, especially for cholesterol and triglycerides. There are three main APOE alleles: ε2, ε3, and ε4. The APOE ε4 allele is significantly associated with higher LDL-C levels, and ε4 carriers are typically susceptible to hypercholesterolemia, with an increased risk of CVD and dementia [29,30]. Collectively, these findings suggest that lipid metabolism pathways may be intricately linked to PD, warranting further investigation to confirm these associations.

Previous studies have shown that diabetes mellitus is an independent risk factor for PD and is also associated with the progression of motor and cognitive symptoms [31]. A large Korean cohort study, which included 17,163,560 individuals, revealed that metabolic syndrome was an independent risk factor for PD, and low high-density lipoprotein cholesterol and hyperglycemia showed the strongest associations with PD risk [32]. However, the present study did not find an association between diabetes mellitus and PD symptom progression. This may be due to the small number of patients with diabetes mellitus in our cohort, so the potential effects of these factors on PD progression cannot be ruled out.

Our study has several strengths. Firstly, we included comprehensive CVD risk factors, and quantified overall CVD risk using both CVD risk points and the CVD risk ratio, a nuanced approach to capturing cardiovascular burden. Secondly, our analysis of motor and non-motor symptoms was highly detailed and stratified: motor symptoms were assessed separately in “on” and “off” states, and individual sub-scores for tremor, bradykinesia, PIGD, axial motor symptoms, psychotic symptoms, mood complaints, sleep disorders, and autonomic symptoms were analyzed. This detailed stratification allowed us to identify subtle associations between specific symptoms and disease progression. Thirdly, brain MRI findings and genetic testing results were integrated, which enhanced the mechanistic depth of our insights into PD pathogenesis. Finally, the study had an extended follow-up duration (mean of 9.2 years; maximum of 14 years), which better reflects the long-term progression of PD. These strengths collectively enhance the robustness and generalizability of our findings.

However, our study has several limitations. Firstly, the research design was constrained by data availability from the PPMI: the sample size was modest, and LDL-C, high-density lipoprotein cholesterol, and triglycerides were excluded from the analysis due to limited serum lipid data within the cohort. This reduces the accuracy of CVD risk estimation in our study. Secondly, we employed a simplified FRS to estimate general CVD risk. While FRS is an early, widely adopted CVD risk assessment tool first proposed nearly 20 years ago, it effectively estimates 10-year coronary heart disease risk [21] but may lack generalizability to contemporary populations. The original Framingham cohort was demographically homogeneous, and geographically restricted, with higher smoking prevalence and lower uptake of preventive therapies compared to contemporary populations [33]. Updated CVD risk assessment models (e.g., PCE [34], WHO CVD score [35], SCORE-2 [36], PREVENT [37,38]) now incorporate additional indicators (e.g., ethnicity, triglycerides, HDL-C, and estimated glomerular filtration rate [eGFR]), and future studies should leverage these tools for more robust CVD risk stratification. Thirdly, only baseline CVD risk factors were analyzed, but baseline measurements do not reflect longitudinal changes in these factors or the cumulative effects of sustained exposure over time. Accordingly, our findings solely demonstrate an association between baseline CVD risk and PD progression, and cannot be construed as causal relationships linked to long-term exposure. Additionally, the interaction between anti-PD medications and CVD risk factors was not examined in detail. PD patients frequently exhibit cardiovascular autonomic dysfunction, and anti-PD agents such as levodopa, dopamine agonists, and anticholinergics may exacerbate this dysfunction and elevate CVD risk. Longitudinal studies with serial measurements of CVD risk factors and medication exposure are required to clarify this potential interactive effect.

In conclusion, this study demonstrates that CVD risk factors are associated with faster motor progression and more severe non-motor symptoms in PD. Our findings provide supporting evidence that targeted interventions for CVD risk factors may slow PD progression, thereby offering new insights into comorbidity management for patients with PD. The association between CVD risk and PD is complex and multifaceted, necessitating additional in-depth research to fully elucidate the underlying mechanisms and clinical implications.

## Figures and Tables

**Table 1:** Demographic and clinical characteristics of PD patients and HCs at baseline.

Characteristics	PD (*n* = 169)	HCs (*n* = 78)	*p* Value
Male, *n* (%)	104 (61.5)	51 (65.4)	0.660
Age, years	61.7 ± 8.1	60.0 ± 10.2	0.057
Smoking, *n* (%)	56 (33.1)	33 (42.3)	0.210
Coronary heart disease, *n* (%)	10 (5.9)	3 (3.8)	0.760
Hypertension, *n* (%)	36 (21.3)	11 (14.1)	0.244
Diabetes, *n* (%)	13 (7.7)	4 (5.1)	0.639
Hyperlipidemia, *n* (%)	41 (24.3)	22 (28.2)	0.614
Cerebral vascular disease, *n* (%)	4 (2.4)	0 (0)	0.311
Gout, *n* (%)	0 (0)	1 (1.3)	0.316
Obesity, *n* (%)	36 (21.3)	18 (23.1)	0.882
Disease duration, years	0.5 (1.0)	/	
Systolic blood pressure (mmHg)	128.7 ± 15.3	127.2 ± 14.7	0.445
Diastolic blood pressure (mmHg)	75.7 ± 9.9	76.0 ± 10.4	0.951
BMI (kg/m^2^)	25.8 (5.8)	26.2 (5.0)	0.102
Education, years	17 (2)	16 (3)	0.286
Follow-up time, years	8.2 (5.0)	11.5 (4.1)	<0.001
Family history, *n* (%)	68 (40.2)	11 (14.1)	<0.001
*ApoE* ε4 status, *n* (%)			
Negative	135 (79.9)	57 (73.1)	0.331
Heterozygous	30 (17.8)	20 (25.6)	
Homozygous	4 (2.4)	1 (1.3)	
Count of pathogenic variants, *n* (%)			
0/1/2	109(64.5)/55(32.5)/5 (3.0)	77 (98.7)/1 (1.3)/0 (0)	<0.001
Brain MRI result, *n* (%)			
Normal	110 (65.1)	57 (73.1)	0.349
Not clinically significantly abnormal	56 (33.1)	19 (24.4)	
Clinically significantly abnormal	3 (1.8)	2 (2.6)	
CVD risk points	14.4 ± 4.7	14.2 ± 4.9	0.062
CVD risk, *n* (%)			
<10%	0	2 (2.6)	0.045
10–20%	52 (30.8)	17 (21.8)	
>20%	117 (69.2)	59 (75.6)	
CVD risk ratio	1.35 ± 0.4	1.37 ± 0.4	0.735
MoCA	28 (3)	28 (2)	0.211
MDS-UPDRS part I	5 (6)	2.5 (3.75)	<0.001
MDS-UPDRS part II	5 (6)	0 (0)	<0.001
MDS-UPDRS part III	20 (13)	0 (2)	<0.001
Axial motor sub-score	2 (2)	0 (0)	<0.001

**Table 2:** Univariate linear regression analyses of the relationships between CVD points, CVD risk, CVD risk ratio and motor symptoms in PD patients.

Motor Symptoms	CVD Risk Points		CVD Risk		CVD Risk Ratio	
	*β*	*p* Value	*β*	*p* Value	*β*	*p* Value
Hoehn–Yahr stage						
Baseline	0.009	0.325	−0.030	0.734	−0.138	0.206
Follow-up in “on” state	0.012	0.207	−0.045	0.641	0.149	0.211
Follow-up in “off” state	0.010	0.352	−0.092	0.403	0.094	0.493
MDS-UPDRS Part II						
Baseline	0.046	0.496	−0.985	0.152	0.633	0.459
Follow-up	0.237	0.060	−1.207	0.347	3.529	0.026
MDS-UPDRS Part III						
Baseline	0.305	0.049	−0.611	0.700	3.412	0.082
Follow-up in “on” state	0.596	0.011	−3.861	0.108	2.482	0.407
Change in “on” state	0.291	0.242	−3.250	0.199	−0.930	0.768
Follow-up in “off” state	0.128	0.651	−1.585	0.582	2.455	0.492
Change in “off” state	−0.177	0.536	−0.974	0.738	−0.957	0.791
Tremor sub-score						
Baseline	0.142	0.014	−0.358	0.544	1.736	0.017
Follow-up in “on” state	0.116	0.047	−0.942	0.115	0.003	0.526
Follow-up in “off” state	0.074	0.478	−0.748	0.479	0.745	0.861
Rigidity sub-score						
Baseline	−0.024	0.592	0.218	0.627	0.091	0.870
Follow-up in “on” state	0.045	0.463	−0.262	0.671	−0.168	0.827
Follow-up in “off” state	−0.047	0.497	−0.226	0.750	−0.622	0.480
Bradykinesia sub-score						
Baseline	0.094	0.291	−0.154	0.865	1.508	0.179
Follow-up in “on” state	0.278	0.020	−1.457	0.235	2.206	0.147
Follow-up in “off” state	−0.043	0.754	−0.417	0.766	1.624	0.350
PIGD sub-score						
Baseline	0.005	0.670	0.152	0.225	−0.095	0.539
Follow-up in “on” state	0.055	0.081	0.038	0.362	0.501	0.212
Follow-up in “off” state	0.040	0.333	−0.295	0.926	0.383	0.458
Axial motor sub-score						
Baseline	0.082	0.002	−0.361	0.184	0.176	0.603
Follow-up in “on” state	0.143	0.009	−1.019	0.071	0.882	0.210
Change in “on” state	0.061	0.249	−0.658	0.221	0.706	0.291
Follow-up in “off” state	0.136	0.024	−0.703	0.254	1.072	0.161
Change in “off” state	0.054	0.349	−0.342	0.557	0.896	0.215
MDS-UPDRS Part IV						
Baseline	0.030	0.331	−0.286	0.355	0.474	0.217
Follow-up	−0.055	0.418	1.274	0.064	0.726	0.397
LEDD						
Follow-up	−5.069	0.553	79.118	0.363	243.356	0.024

All presented *p*-values are unadjusted raw values. The significance threshold after Bonferroni correction for multiple comparisons was set at *p* < 0.05. No associations remained statistically significant after Bonferroni correction (all adjusted *p* > 0.05).

**Table 3:** Univariate linear regression analyses of the relationships between CVD risk points, CVD risk, CVD risk ratio and non-motor symptoms in PD patients.

Non-Motor Symptoms	CVD Risk Points		CVD Risk		CVD Risk Ratio	
	*β*	*p* Value	*β*	*p* Value	*β*	*p* Value
MDS-UPDRS Part I						
Baseline	0.108	0.099	−1.472	0.027	0.865	0.298
Follow-up	0.197	0.038	−1.824	0.060	1.538	0.203
Psychotic symptom sub-score						
Baseline	0.004	0.276	−0.051	0.154	0.050	0.264
Follow-up	0.016	0.062	−0.090	0.298	0.135	0.206
Mood complaint sub-score						
Baseline	−0.030	0.128	−0.297	0.134	−0.035	0.889
Follow-up	0.008	0.817	−0.562	0.089	−0.071	0.862
Sleep disorder sub-score						
Baseline	0.061	0.016	−0.432	0.095	0.519	0.107
Follow-up	0.037	0.188	−0.150	0.599	0.440	0.212
Autonomic symptom sub-score						
Baseline	0.048	0.007	−0.380	0.036	−0.083	0.715
Follow-up	0.027	0.282	−0.333	0.184	−0.130	0.678
MoCA						
Baseline	−0.134	<0.001	0.105	0.767	−0.677	0.475
Follow-up	−0.208	**<0.001**	0.615	0.244	−0.558	0.708

All presented *p*-values are unadjusted raw values. The significance threshold after Bonferroni correction for multiple comparisons was set at *p* < 0.05. *p*-values corresponding to associations that remain statistically significant after correction are formatted in bold.

**Table 4:** Demographic and clinical characteristics of moderate-to-low- and high-CVD-risk PD patients.

Characteristics	Moderate-to-Low CVD Risk (*n* = 52)	High CVD Risk (*n* = 117)	*p* Value
Male, *n* (%)	22 (42.3)	82 (70.1)	0.001
Age, years	57.9 ± 6.8	63.3 ± 8.1	<0.001
Smoking, *n* (%)	10 (19.2)	46 (39.3)	0.017
Coronary heart disease, *n* (%)	3 (5.8)	7 (6.0)	1
Hypertension, *n* (%)	6 (11.5)	30 (25.6)	0.062
Diabetes, *n* (%)	2 (3.8)	11 (9.4)	0.348
Hyperlipidemia, *n* (%)	8 (15.4)	33 (28.2)	0.110
Cerebral vascular disease, *n* (%)	0 (0)	1 (3.4)	0.313
Obesity, *n* (%)	12 (23.1)	24 (20.5)	0.863
Disease duration, years	0.4 (0.9)	0.6 (1.2)	0.490
Systolic blood pressure (mmHg)	125.7 ± 13.3	129.2 ± 15.8	0.074
Diastolic blood pressure (mmHg)	73.7 ± 9.4	76.5 ± 10.1	0.079
BMI (kg/m^2^)	25.3 (6.1)	26.6 (5.3)	0.564
Education, years	16 (3)	17 (3)	0.054
Follow-up time, years	9.5 (5.0)	8.1 (4.4)	0.369
Family history, *n* (%)	23 (44.2)	45 (38.5)	0.592
*ApoE* ε4 status, n (%)			
Negative	42 (80.8)	93 (79.5)	0.654
Heterozygous	8 (1.4)	22 (18.8)	
Homozygous	2 (3.8)	2 (1.7)	
Count of pathogenic variants, *n* (%)			
0/1/2	35 (67.3)/16 (30.8)/1 (1.9)	74 (63.2)/39 (33.3)/4 (3.4)	0.902
Brain MRI result, *n* (%)			
Normal	35 (67.3)	75 (64.1)	0.309
Not clinically significantly abnormal	15 (28.8)	41 (35.0)	
Clinically significantly abnormal	2 (3.8)	1 (0.9)	
Motor subtypes, *n* (%)			
TD	37 (71.2)	86 (73.5)	0.676
Indeterminate	4 (7.7)	12 (10.3)	
PIGD	11 (21.2)	19 (16.2)	
CVD points	12 ± 4.3	16 ± 6.0	<0.001
CVD risk ratio	1.21 ± 0.4	1.33 ± 0.4	0.035

**Table 5:** Motor symptoms of subgroups of high- and moderate-to-low-CVD risk PD patients.

Motor Symptoms	Moderate-to-Low CVD Risk (*n* = 52)	High CVD Risk (*n* = 117)	*p* Value
Hoehn–Yahr stage, *n* (%)			
Baseline 1/2/3	23 (44.2)/28 (53.8)/1 (1.9)	48 (41.0)/67 (57.3)/2 (1.7)	0.846
Follow-up in “on” state 1/2/3/4	4 (7.7)/43 (82.7)/3 (5.8)/2 (3.8)	8 (6.9)/93 (79.5)/11 (9.4)/5 (4.3)	0.681
Follow-up in “off” state 1/2/3/4/5	2 (3.8)/41 (78.8)/7 (13.5)/ 2 (3.8)/0 (0)	3 (2.6)/92 (78.6)/12 (10.3)/ 8 (6.8)/2 (1.7)	0.724
MDS-UPDRS Part II			
Baseline	5 (3.25)	5 (6)	0.411
Follow-up	10.5 (7.25)	12 (10)	0.260
MDS-UPDRS Part III			
Baseline	18 (16)	20 (11)	0.522
Follow-up in “on” state	21.5 (16.5)	25 (21)	0.132
Change in “on” state	2.5 (14)	6 (20)	0.348
Follow-up in “off” state	39.5 (20.5)	38 (24)	0.827
Change in “off” state	18.5 (18.5)	17 (22.5)	0.784
Tremor sub-score			
Baseline	4 (4.25)	4 (5)	0.467
Follow-up in “on” state	1 (4)	2 (4)	0.782
Follow-up in “off” state	6 (8.25)	6 (7)	0.350
Rigidity sub-score			
Baseline	3 (4)	3(3)	0.782
Follow-up in “on” state	3.5 (5.25)	4 (5)	0.363
Follow-up in “off” state	8 (6.25)	6 (7)	0.485
Bradykinesia sub-score			
Baseline	8 (8.5)	8 (7)	0.692
Follow-up in “on” state	11 (8.5)	12 (12)	0.260
Follow-up in “off” state	17 (10.25)	17 (11)	0.769
PIGD sub-score			
Baseline	1 (1)	1 (1)	0.119
Follow-up in “on” state	1 (2)	1 (1)	0.157
Follow-up in “off” state	2 (3)	2 (2)	0.704
Axial motor sub-score			
Baseline	1 (1)	2 (2)	0.136
Follow-up in “on” state	3 (4)	4 (5)	0.048
Change in “on” state	1 (4)	3 (3)	0.132
Follow-up in “off” state	5 (4.25)	5 (5)	0.370
Change in “off” state	3 (5)	4 (4)	0.469
MDS-UPDRS Part IV			
Baseline	0 (0)	0 (0)	0.335
Follow-up	5 (5)	3 (6)	0.018
LEDD			
Follow-up	690 (619.9)	600 (893)	0.297

**Table 6:** Non-motor symptoms of subgroups of moderate-to-low- and high-CVD-risk PD patients.

Non-Motor Symptoms	Moderate-to-Low CVD Risk (*n* = 52)	High CVD Risk (*n* = 117)	*p* Value
MDS-UPDRS Part I			
Baseline	4 (4.25)	5 (6)	0.082
Follow-up	7 (6.25)	9 (7)	0.039
Psychotic symptom sub-score			
Baseline	0 (0)	0 (0)	0.647
Follow-up	0 (0)	0 (0)	0.369
Mood complaint sub-score			
Baseline	0 (1)	0 (1)	0.424
Follow-up	0 (2)	1 (3)	0.021
Sleep disorder sub-score			
Baseline	1 (2.25)	2 (2)	0.097
Follow-up	2.5 (3)	2 (3)	0.648
Autonomic symptom sub-score			
Baseline	0 (1)	1 (2)	0.058
Follow-up	1 (2)	1 (3)	0.184
MoCA			
Baseline	28 (3)	27 (3)	0.612
Follow-up	28 (3)	27 (4)	0.112

## Data Availability

Data are available from the corresponding author upon reasonable request.

## References

[1] Su D., Cui Y., He C., Yin P., Bai R., Zhu J., Lam J.S.T., Zhang J., Yan R., Zheng X. (2025). Projections for prevalence of Parkinson’s disease and its driving factors in 195 countries and territories to 2050: Modelling study of Global Burden of Disease Study 2021. BMJ.

[2] Ben-Shlomo Y., Darweesh S., Llibre-Guerra J., Marras C., San Luciano M., Tanner C. (2024). The epidemiology of Parkinson’s disease. Lancet.

[3] Tanner C.M., Ostrem J.L. (2024). Parkinson’s Disease. N. Engl. J. Med..

[4] Del Din S., Elshehabi M., Galna B., Hobert M.A., Warmerdam E., Suenkel U., Brockmann K., Metzger F., Hansen C., Berg D. (2019). Gait analysis with wearables predicts conversion to Parkinson disease. Ann. Neurol..

[5] Bloem B.R., Okun M.S., Klein C. (2021). Parkinson’s disease. Lancet.

[6] Madetko-Alster N., Alster P. (2025). Importance of hypoglycemia episodes and glycemic variability in Parkinsonian syndromes. Neurol. Neurochir. Pol..

[7] Martin S.S., Aday A.W., Almarzooq Z.I., Anderson C.A., Arora P., Avery C.L., Baker-Smith C.M., Gibbs B.B., Beaton A.Z., Boehme A.K. (2024). 2024 Heart Disease and Stroke Statistics: A Report of US and Global Data from the American Heart Association. Circulation.

[8] Alves M., Caldeira D., Ferro J.M., Ferreira J.J. (2020). Does Parkinson’s disease increase the risk of cardiovascular events? A systematic review and meta-analysis. Eur. J. Neurol..

[9] Lange L.M., Cerquera-Cleves C., Schipper M., Panagiotaropoulou G., Braun A., Kraft J., Awasthi S., Bell N., Posthuma D., Ripke S. (2025). Prioritizing Parkinson’s disease risk genes in genome-wide association loci. npj Parkinson’s Dis..

[10] Al-Kuraishy H.M., Al-Hamash S.M., Jabir M.S., Al-Gareeb A.I., Albuhadily A.K., Albukhaty S., Sulaiman G.M. (2024). The classical and non-classical axes of renin-angiotensin system in Parkinson disease: The bright and dark side of the moon. Ageing Res. Rev..

[11] Ke L., Zhao L., Xing W., Tang Q. (2024). Association between Parkinson’s disease and cardiovascular disease mortality: A prospective population-based study from NHANES. Lipids Health Dis..

[12] Acharya S., Lumley A.I., Devaux Y., NCER-PD Consortium (2024). Cardiovascular history and risk of idiopathic Parkinson’s disease: A cross-sectional observational study. BMC Neurosci..

[13] Leshchinski T., Rozani V., Giladi N., Bitan M., Peretz C. (2023). Incidence of cardiovascular morbidity among Parkinson’s disease patients; a large-scale cohort study in a 16-year time window around disease onset. Park. Relat. Disord..

[14] Kotagal V., Albin R.L., Müller M.L.T.M., Koeppe R.A., Frey K.A., Bohnen N.I. (2014). Modifiable cardiovascular risk factors and axial motor impairments in Parkinson disease. Neurology.

[15] Oosterwegel M.J., Krijthe J.H., den Brok M.G.H.E., van den Heuvel L., Richard E., Heskes T., Bloem B.R., Evers L.J.W. (2023). The effect of cardiovascular risk on disease progression in de novo Parkinson’s disease patients: An observational analysis. Front. Neurol..

[16] Carroll C.B., Wyse R.K.H. (2017). Simvastatin as a Potential Disease-Modifying Therapy for Patients with Parkinson’s Disease: Rationale for Clinical Trial, and Current Progress. J. Parkinson’s Dis..

[17] Stevens K.N., Creanor S., Jeffery A., Whone A., Zajicek J., Foggo A., Jones B., Chapman R., Cocking L., Wilks J. (2022). Evaluation of Simvastatin as a Disease-Modifying Treatment for Patients with Parkinson Disease: A Randomized Clinical Trial. JAMA Neurol..

[18] (2011). Parkinson Progression Marker Initiative. The Parkinson Progression Marker Initiative (PPMI). Prog. Neurobiol..

[19] Jost S.T., Kaldenbach M.-A., Antonini A., Martinez-Martin P., Timmermann L., Odin P., Katzenschlager R., Borgohain R., Fasano A., Stocchi F. (2023). Levodopa Dose Equivalency in Parkinson’s Disease: Updated Systematic Review and Proposals. Mov. Disord..

[20] GBD 2021 US Obesity Forecasting Collaborators (2024). National-level and state-level prevalence of overweight and obesity among children, adolescents, and adults in the USA, 1990–2021, and forecasts up to 2050. Lancet.

[21] D’Agostino R.B., Vasan R.S., Pencina M.J., Wolf P.A., Cobain M., Massaro J.M., Kannel W.B. (2008). General cardiovascular risk profile for use in primary care: The Framingham Heart Study. Circulation.

[22] Nordet P., Mendis S., Dueñas A., de la Noval R., Armas N., De La Noval I.L., Pupo H. (2013). Total cardiovascular risk assessment and management using two prediction tools, with and without blood cholesterol. MEDICC Rev..

[23] Grant J.K., Ndumele C.E., Martin S.S. (2024). The Evolving Landscape of Cardiovascular Risk Assessment. JAMA.

[24] Stebbins G.T., Goetz C.G., Burn D.J., Jankovic J., Khoo T.K., Tilley B.C. (2013). How to identify tremor dominant and postural instability/gait difficulty groups with the movement disorder society unified Parkinson’s disease rating scale: Comparison with the unified Parkinson’s disease rating scale. Mov. Disord..

[25] Hu G. (2010). Total cholesterol and the risk of Parkinson’s disease: A review for some new findings. Parkinson’s Dis..

[26] Gudala K., Bansal D., Muthyala H. (2013). Role of serum cholesterol in Parkinson’s disease: A meta-analysis of evidence. J. Parkinson’s Dis..

[27] Rozani V., Gurevich T., Giladi N., El-Ad B., Tsamir J., Hemo B., Peretz C. (2018). Higher serum cholesterol and decreased Parkinson’s disease risk: A statin-free cohort study. Mov. Disord..

[28] Dai L., Wang J., Zhang X., Yan M., Zhou L., Zhang G., Meng L., Chen L., Cao X., Zhang Z. (2023). 27-Hydroxycholesterol Drives the Spread of α-Synuclein Pathology in Parkinson’s Disease. Mov. Disord..

[29] Raulin A.-C., Doss S.V., Trottier Z.A., Ikezu T.C., Bu G., Liu C.-C. (2022). ApoE in Alzheimer’s disease: Pathophysiology and therapeutic strategies. Mol. Neurodegener..

[30] Huebbe P., Rimbach G. (2017). Evolution of human apolipoprotein E (APOE) isoforms: Gene structure, protein function and interaction with dietary factors. Ageing Res. Rev..

[31] Chohan H., Senkevich K., Patel R.K., Bestwick J.P., Jacobs B.M., Ciga S.B., Gan-Or Z., Noyce A.J. (2021). Type 2 Diabetes as a Determinant of Parkinson’s Disease Risk and Progression. Mov. Disord..

[32] Nam G.E., Kim S.M., Han K., Kim N.H., Chung H.S., Kim J.W., Han B., Cho S.J., Yu J.H., Park Y.G. (2018). Metabolic syndrome and risk of Parkinson disease: A nationwide cohort study. PLoS Med..

[33] Gaziano T.A., Gaziano J.M. (2024). Can Cardiovascular Risk Assessment Be Improved in the 21st Century?. JAMA.

[34] Goff D.C., Lloyd-Jones D.M., Bennett G., Coady S., D’Agostino R.B., Gibbons R., Greenland P., Lackland D.T., Levy D., O’Donnell C.J. (2014). 2013 ACC/AHA guideline on the assessment of cardiovascular risk: A report of the American College of Cardiology/American Heart Association Task Force on Practice Guidelines. Circulation.

[35] Kaptoge S., Pennells L., De Bacquer D., Cooney M.T., Kavousi M., Stevens G., Riley L.M., Savin S., Khan T., Altay S. (2019). World Health Organization cardiovascular disease risk charts: Revised models to estimate risk in 21 global regions. Lancet Glob. Health.

[36] SCORE2 Working Group and ESC Cardiovascular Risk Collaboration (2021). SCORE2 risk prediction algorithms: New models to estimate 10-year risk of cardiovascular disease in Europe. Eur. Heart J..

[37] Khan S.S., Matsushita K., Sang Y., Ballew S.H., Grams M.E., Surapaneni A., Blaha M.J., Carson A.P., Chang A.R., Ciemins E. (2024). Development and Validation of the American Heart Association’s PREVENT Equations. Circulation.

[38] Diao J.A., Shi I., Murthy V.L., Buckley T.A., Patel C.J., Pierson E., Yeh R.W., Kazi D.S., Wadhera R.K., Manrai A.K. (2024). Projected Changes in Statin and Antihypertensive Therapy Eligibility with the AHA PREVENT Cardiovascular Risk Equations. JAMA.

